# CAN VIRTUAL EXERCISE PROMOTE PHYSICAL RESILIENCE DURING THE COVID-19 PANDEMIC AMONG ACTIVE OLDER ADULTS?

**DOI:** 10.1093/geroni/igac059.1631

**Published:** 2022-12-20

**Authors:** Kenneth Manning, Stephen Jennings, Megan Pearson, Richard Sloane, Katherine Hall, Kyle Bourassa, Miriam Morey

**Affiliations:** Durham VA Healthcare System, Durham, North Carolina, United States; Durham VA Healthcare System, Durham, North Carolina, United States; Durham VA Healthcare System, Durham, North Carolina, United States; Duke University Medical Center, Durham, North Carolina, United States; Durham VA Health Care System, Durham, North Carolina, United States; Durham VA Healthcare System, Durham, North Carolina, United States; Durham VA Health Care System, Durham, North Carolina, United States

## Abstract

**Background:**

Gerofit is a facility-based exercise and health promotion program for older Veterans that transitioned to virtual delivery in March 2020. Little is known about how virtual exercise would promote resilience in the physical function of individuals previously participating in-person.

**Methods:**

Preliminary data from 1 of 14 sites was gathered 72 Veterans returning to facility-based exercise after COVID mandated shutdowns. 39 individuals chose not to participate virtually, and 33 actively participated virtually for over 1 year. Re-entry data were then compared to the patients’ most recent test. Assessment means were compared within groups.

**Results:**

Change scores from T1 to T2 were: -1.19 versus +2.4 repetitions for 30-second arm curls; +1.57 repetitions for 30-second chair stands; and -113.87 versus -77.3 yards for six-minute walk distance for non-virtual versus virtual groups. Implications: Participation in virtual exercise interventions may promote resilience and resistance to functional decline in previously active individuals during enforced isolation.

